# Polarization-Controlled Femtosecond Laser Texturing Enables Robust Antifouling Stainless Steel Surfaces

**DOI:** 10.3390/molecules31030480

**Published:** 2026-01-29

**Authors:** Eunyeop Ji, Daesik Ko, Chan Hyeon Yang, Vassilia Zorba, Jung Hwan Park, Kyueui Lee, Minok Park

**Affiliations:** 1 Department of Mechanical and Automotive Engineering, Kongju National University, Cheonan 31080, Republic of Korea; 2 Department of Chemistry, Kyungpook National University, Daegu 41566, Republic of Korea; 3 Department of Chemistry Education, Kyungpook National University, Daegu 41566, Republic of Korea; 4 Department of Mechanical Engineering (Department of Aeronautics, Mechanical and Electronic Convergence Engineering), Kumoh National Institute of Technology, 61, Daehak-ro, Gumi, Gyeongbuk 39177, Republic of Korea; 5 Energy Technologies Area, Lawrence Berkeley National Laboratory, Berkeley, CA 94720, USA; 6 KNU G-LAMP Project Group and KNU Institute of Basic Sciences, Kyungpook National University, Daegu 41566, Republic of Korea; 7 Biomedical Research Institute, Kyungpook National University Hospital, Daegu 41940, Republic of Korea

**Keywords:** femtosecond laser processing, antifouling, laser-induced periodic surface structures, contact angles

## Abstract

In this work, we demonstrate precise control over laser-induced periodic surface structures (LIPSS) on stainless steel (SS) using femtosecond (fs) laser processing to suppress bacterial adhesion. We systematically compare the antifouling behavior of laser-textured surfaces with distinct pattern directionalities—linear and circular. Fs laser irradiation with linear polarization produces directional and anisotropic LIPSS, which progressively evolve into more complex hierarchical surface textures as processing conditions vary. In contrast, fs laser irradiation with circular polarization yields isotropic surface morphologies. Despite these morphological differences, the surface wettability remains nearly constant, with contact angles confined to a narrow range of 32.6–36.9°. Bacterial adhesion tests using *Escherichia coli* reveal that surfaces patterned with anisotropic features generated by linear polarization—particularly at an incident power of 30 mW—exhibit enhanced antifouling performance compared to isotropic counterparts. These results indicate that antifouling efficacy is governed not only by surface wettability but also by the spatial organization and anisotropy of the LIPSS. This study highlights the critical role of polarization-controlled fs laser processing in tailoring surface architectures and provides a rational strategy for designing bio-resistant metallic surfaces.

## 1. Introduction

Biofouling—the unwanted accumulation of microorganisms, proteins, and organic matter—presents a critical challenge across industries relying on stainless steel (SS) infrastructure [1]. For example, in marine engineering, biofouling increases drag in ship hulls and accelerates microbiologically influenced corrosion on pipelines [2,3]. Similarly, in healthcare and food processing, the adhesion of pathogens to surgical instruments and hygiene lines creates severe contamination risks [4,5,6]. Although SS is the standard material for these applications due to its mechanical robustness, its naturally high surface energy facilitates bacterial colonization [7,8].

Various strategies have been employed to mitigate biofouling on metallic surfaces. For instance, polymer-based fouling-release coatings, such as polyethylene glycol and silicone elastomers, have been explored to reduce adhesion through low surface energy and hydration layers [9]. Moreover, biomimetic surface texturing inspired by shark skin and mollusk shells has attracted attention for its potential to physically deter bacterial attachment.

While these non-toxic methods offer improved environmental compatibility, they still face significant limitations. Specifically, conventional polymer coatings, such as polydopamine films [10], often suffer from limited mechanical durability and are prone to delamination under abrasive conditions, requiring frequent reapplication [11,12]. In addition, conventional surface roughening or machining processes provide a scalable means to alter surface topography but lack the spatial resolution needed to create well-defined micro/nanostructures and can introduce uncontrolled, stochastic micro-defects that serve as bacterial anchoring sites [13,14]. Therefore, there is a pressing need for non-toxic, precise, and durable surface engineering strategies that can provide intrinsic antifouling performance without relying on hazardous chemical agents or imprecise mechanical processes [15].

Femtosecond (fs) laser texturing has emerged as a powerful alternative to conventional stochastic roughening methods and organic coatings. It enables highly controlled and reproducible surface morphologies that are difficult to achieve with traditional approaches [16,17,18]. Moreover, unlike additive coatings, this technique creates hierarchical structures by directly restructuring the bulk material. This monolithic nature ensures that the resulting surface features are intrinsically integrated into the substrate, offering superior resistance to delamination and mechanical wear [19]. Furthermore, in contrast to nanosecond [20] or continuous-wave lasers, where prolonged thermal diffusion often limits structural precision, fs laser processing enables the precise fabrication of hierarchical micro/nanostructures [21]. This capability arises because the energy deposition time is significantly shorter than the electron–phonon relaxation time in metals, which is typically on the order of a few picoseconds [22]. As a result, fs laser irradiation can generate a wide range of surface features, spanning from laser-induced periodic surface structures (LIPSS) to deep micro-grooves [23], which are essential for controlling wettability [24] and mechanical durability.

Regarding fs laser surface engineering, previous studies have widely demonstrated the potential of laser-fabricated surfaces to repel bacteria by minimizing the effective contact area available for cell anchorage [25]. Research utilizing LIPSS has particularly highlighted that these nanoscale features can act as physical barriers, disrupting initial bacterial adhesion [26,27]. However, relying solely on static contact angles (i.e., wettability) is often insufficient for long-term biofouling resistance [28,29,30]. Moreover, most existing fs laser studies have focused primarily on optimizing wettability through simple surface topographies [24]. Consequently, there remains a lack of comprehensive research on how diverse surface morphologies induced by different polarization states specifically influence bacterial behavior.

In this work, we fabricated hierarchically textured SS surfaces by manipulating laser polarization using a 500-fs laser system to systematically evaluate their antifouling performance. The antifouling performance of laser-textured surfaces with different pattern directionality, specifically linear and circular geometries, was systematically compared. Moving beyond simple wettability metrics, we elucidated the critical role of specific polarization-dependent micro-/nano-pattern geometries in suppressing biofilm formation. Most notably, our optimized laser-textured surfaces demonstrated exceptional antibacterial efficacy, significantly reducing bacterial survival compared to untreated counterparts. This study not only highlights the outstanding capability of fs laser processing in creating durable antifouling interfaces but also provides a practical framework for mitigating biofouling in industrial environments.

## 2. Results

### 2.1. Femtosecond Laser Processing

The fs laser texturing was employed to tailor the surface morphology of SS, enabling precise and reproducible control over micro- and nanoscale surface structures that are critical for functional surface engineering. The ultrashort pulse duration of the fs laser minimizes thermal diffusion into the substrate, thereby allowing localized material modification while suppressing excessive melting or heat-affected zones. This characteristic makes fs laser processing particularly suitable for generating hierarchical surface structures on metallic substrates.

**Figure 1:**
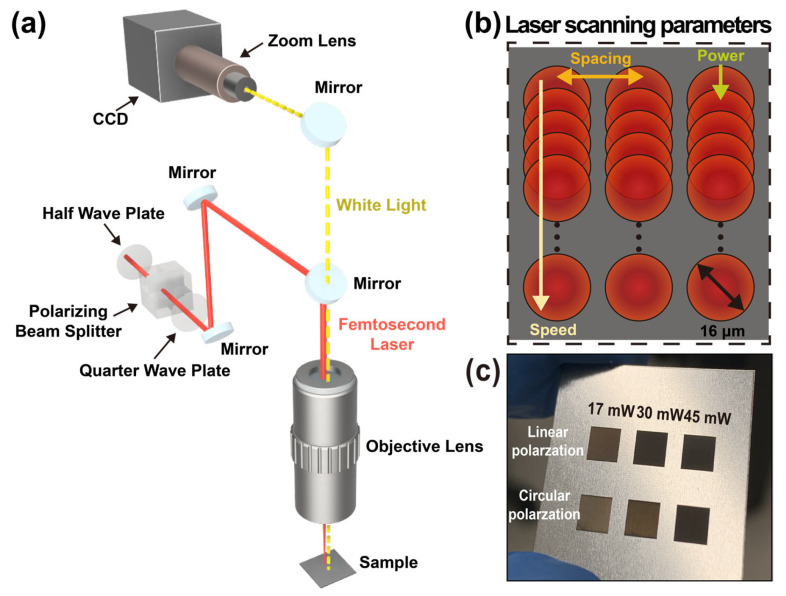
Schematic of the fs laser texturing system and surface processing strategy. (**a**) Schematic illustration of the fs laser surface texturing setup, including laser power control using a half-wave plate and polarizing beam splitter, polarization tuning via a quarter-wave plate, and in situ monitoring with a co-illumination imaging system. The fs laser beam is focused onto the SS substrate using a microscopic objective lens for high-resolution surface modification. (**b**) Schematic representation of the laser scanning strategy and key processing. While the focused fs laser beam is spatially scanned continuously across the surface at a constant speed of 5 mm/s, the laser–matter interaction is temporally discontinuous due to the ultrashort pulsed nature (100 kHz). The scan lines were spaced by 2 μm to ensure sufficient pulse overlap for uniform surface modification. (**c**) Photographs of SS samples after fs laser processing under different laser power and polarization conditions, demonstrating the tunability of surface morphology achieved through systematic control of laser parameters.

The optical configuration used in this study is schematically illustrated in Figure 1a. A 500-fs laser (1030 nm, 100 kHz) was used for surface patterning, and the beam was focused onto the sample using a 5× near-infrared objective lens to achieve a spot diameter of approximately 16 μm. The fs laser beam was first directed through a half-wave plate in combination with a polarizing beam splitter, which enabled continuous and precise adjustment of the incident laser power without altering other beam characteristics. A quarter-wave plate was subsequently introduced to control the polarization state of the laser beam, allowing systematic tuning between linear and circular polarization. This polarization control was employed to investigate its influence on laser–matter interaction and the resulting surface morphology. The laser beam was then focused onto the SS substrate using a microscopic objective lens, providing a tightly confined focal spot suitable for high-resolution surface texturing. During laser processing, a co-illumination and imaging system was integrated into the experimental setup to facilitate real-time monitoring of surface modification. This system consisted of a white light source, a zoom lens, and a charge-coupled-device camera, which together enabled in situ visualization of the laser-irradiated region during scanning.

The laser scanning strategy and key processing parameters employed in this study are schematically illustrated in Figure 1b. Surface texturing was performed using a raster scanning method, in which the laser beam was scanned line by line across the SS surface. To ensure the reproducibility of the fabrication process, all detailed laser processing parameters, including pulse overlap, the number of scans, and the calculated average fluence for each condition, are listed in Table 1. No assist gas was used. Surface texturing was carried out at a scanning speed of 5 mm/s with a line spacing of 2 μm. These baseline parameters were selected to ensure sufficient overlap between neighboring scan lines, leading to uniform surface modification while maintaining stable processing conditions.

Figure 1c shows photographs of the SS samples after fs laser processing under various laser power and polarization conditions, demonstrating the tunability of surface morphology achievable through controlled adjustment of laser parameters. The observed variations in surface appearance reflect the strong dependence of laser-induced micro- and nanostructure formation on both the delivered energy density and the polarization state of the incident laser beam.

### 2.2. Surface Morphology Characterizations

**Figure 2:**
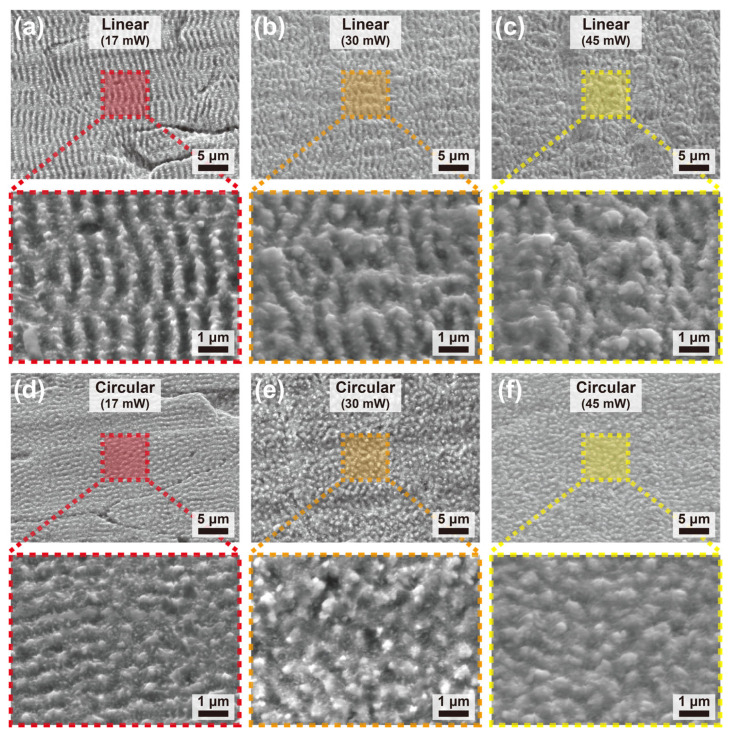
SEM images of femtosecond laser–textured SS surfaces under different laser powers and polarization states. Surfaces processed under linear polarization at laser powers of (**a**) 17 mW, (**b**) 30 mW, and (**c**) 45 mW, respectively, showing the evolution from well-defined, anisotropic ripple-like structures to increasingly irregular and coarsened surface morphologies with increasing laser power. Surfaces processed under circular polarization at laser powers of (**d**) 17 mW, (**e**) 30 mW, and (**f**) 45 mW, respectively, exhibiting fine micro- and nanostructures with weak directional features at low power and densely packed, isotropic surface textures at higher powers.

Figure 2 presents scanning electron microscopy (SEM) images of SS surfaces after fs laser processing under different laser powers and polarization states, highlighting the strong dependence of surface morphology on both parameters. Distinct micro- and nanoscale features are observed across all conditions, indicating effective surface modification induced by fs laser irradiation. Furthermore, surface oxidation is anticipated on the treated surfaces due to the ambient air processing conditions [21].

Under linear polarization (Figure 2a–c), well-defined ripple-like surface structures are formed at a laser power of 17 mW. These structures exhibit a pronounced anisotropic morphology with a preferred orientation, which is characteristic of LIPSS arising from the interference between the incident laser beam and surface-scattered waves. As the laser power increases to 30 mW and 45 mW, the ripple features become progressively less distinct, and the surface morphology transitions toward a more irregular and coarsened texture. This morphological evolution can be attributed to increased energy deposition and enhanced material removal at higher laser powers, which disrupt the periodic ripple formation and promote localized melting and re-solidification.

In contrast, surfaces processed under circular polarization (Figure 2d–f) exhibit markedly different morphological characteristics. At a laser power of 17 mW, fine micro- and nanostructures are formed with weak directional features, reflecting the isotropic energy distribution associated with circularly polarized irradiation. As the laser power is increased to 30 mW and 45 mW, the surface evolves into densely packed textures with reduced directional definition and increased feature density. The absence of a preferred orientation under circular polarization suppresses the formation of strongly anisotropic ripple patterns, resulting instead in more uniform, isotropic surface morphologies governed primarily by laser fluence and cumulative pulse overlap.

These results demonstrate that laser polarization plays a critical role in determining the symmetry and anisotropy of fs laser-induced surface structures, while laser power predominantly governs the degree of material removal and surface coarsening. In principle, increasing the treatment time or pulse repetition frequency increases the number of incident fs laser pulses per unit area, thereby raising the total irradiation dose. As this cumulative irradiation increases, a greater amount of material is removed from the substrate, which drives a morphological transition in the surface topography from nanoscale features (LIPSS, Figure 2a,d) to coarser hierarchical microstructures due to enhanced material removal and local melting (Figure 2c,f). The combined control of laser power and polarization therefore provides a versatile parameter space for tailoring surface morphology on SS, which is essential for optimizing subsequent functional properties such as wettability and antifouling performance.

**Figure 3:**
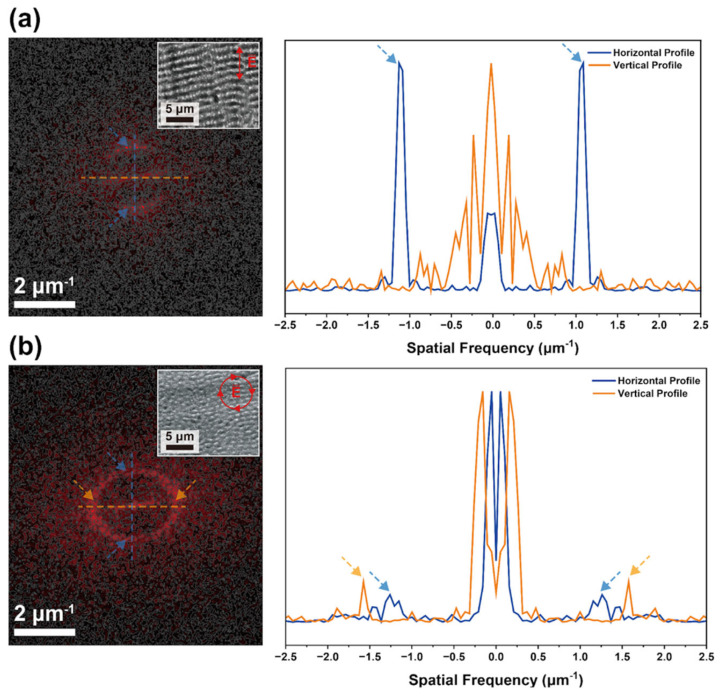
Two-dimensional FFT analysis based on low-magnification SEM images of fs laser–textured SS surfaces processed under (**a**) linear and (**b**) circular polarization. The left panels (**a**,**b**) show the corresponding 2D FFT maps, where the map in (**a**) exhibits a directionally elongated intensity distribution, whereas the map in (**b**) shows a near-circular, rotationally symmetric pattern. The right panels show the spatial-frequency profiles extracted along the horizontal and vertical axes of each FFT map, revealing anisotropic frequency peaks for the linearly polarized surface and broad, isotropic frequency distributions for the circularly polarized surface.

To quantitatively distinguish the surface morphologies observed in the low-magnification SEM images in Figure 2, two-dimensional fast Fourier transform (2D FFT) analysis was performed, as shown in Figure 3, which enables frequency-domain evaluation of spatial periodicity and symmetry and allows a quantitative assessment of surface isotropy and anisotropy.

As shown in Figure 3a, the surface processed under linearly polarized fs laser irradiation exhibits a pronounced anisotropic FFT intensity distribution. The FFT map shows elongated features along a specific direction, indicating the presence of preferentially aligned periodic structures. This anisotropy is evident in the spatial-frequency profile, where the horizontal axis exhibits distinct peaks at approximately ±0.85~0.95 μm^−1^ (Λ: 1.1~1.2 μm), while the vertical axis lacks comparable features. Because spatial frequency quantifies how often a surface pattern repeats per unit length, the appearance of peaks along only one axis confirms the presence of well-defined periodic ordering in a single direction.

In contrast, the surface fabricated under circularly polarized laser irradiation exhibits a nearly rotationally symmetric FFT pattern, as shown in Figure 3b. The corresponding horizontal and vertical spatial-frequency profiles overlap closely, displaying broad, low-amplitude peaks near approximately ±1.5~1.6 μm^−1^ (Λ: 0.6~0.7 μm) rather than sharp, well-defined directional components. This behavior is consistent with the near-circular FFT distribution, indicating isotropic surface organization.

### 2.3. Contact Angle Measurements

**Figure 4:**
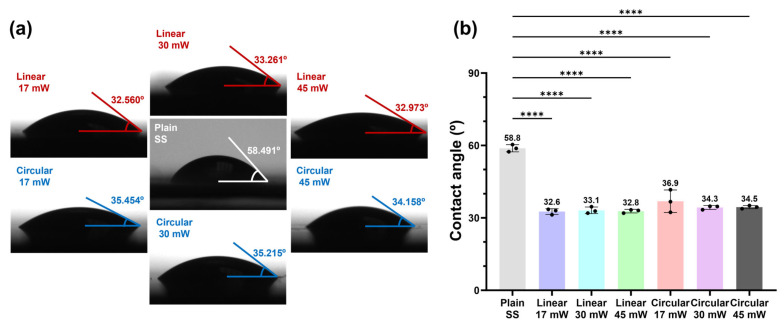
Wettability of untreated SS and fs laser-textured SS surfaces with different LIPSS. (**a**) Representative water contact angle images with superimposed tangent lines. All fs laser-textured surfaces exhibited a consistent contact angle range (32.6–36.9°), regardless of the polarization. All values in the images are representative measurements of contact angle. (**b**) Summary of contact angle distributions for the six LIPSS conditions, indicating a narrow wettability range of approximately 32–36°, consistent with similar surface morphologies. All values indicated above the graph are average values of contact angles. (****: *p* < 0.001; n = 3).

The water contact angles of six different LIPSS conditions were measured to evaluate the wettability of the fs laser–textured SS surfaces, as shown in Figure 4a. Compared to the untreated SS surface (58.8°), all fs laser–textured samples exhibit a pronounced reduction in water contact angle (<37°), indicating enhanced hydrophilicity. The observed hydrophilicity is attributed to the Wenzel model, where laser-induced roughness amplifies the intrinsic hydrophilic nature of the metallic surface. According to Wenzel’s theory [31], the increased effective surface area leads to a lower apparent contact angle on a naturally hydrophilic substrate. This roughness-driven wetting state is distinct from the directional anisotropy effects focused on in our study.

Meanwhile, as shown in Figure 4b, the water contact angles for all six LIPSS conditions are tightly clustered in the range of 32.6–36.9°, indicating only small variations in wettability among the processed surfaces. Notably, this similarity is maintained despite varying the laser power from 17 to 45 mW and changing the polarization state (linear versus circular). Although SEM images (Figure 2) reveal power- and polarization-dependent differences in ripple definition, anisotropy, and texture compactness, the resulting hierarchical micro/nanotextures appear to yield comparable macroscopic wetting responses within the investigated parameter window.

### 2.4. Antifouling Properties

**Figure 5:**
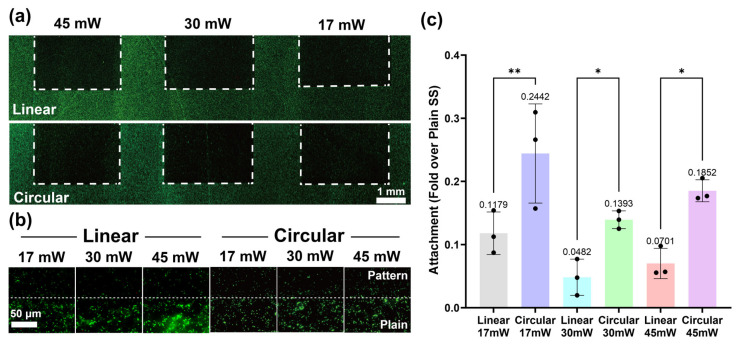
Antifouling effects of LIPSS. (**a**) Antifouling test fluorescence images for 6 LIPSS samples. (**b**) Comparison antifouling performance of non-processed surface and LIPSS at high magnification. (**c**) Summary of antifouling test distributions for the six LIPSS conditions. (*: *p* < 0.05, **: *p* < 0.01; n = 3, pristine surface is the normalization baseline).

Next, we performed the antifouling evaluation using *Escherichia coli* (*E. coli*), as shown in Figure 5a,b. *E. coli* was cultured in Luria–Bertani (LB) broth, harvested by centrifugation, and resuspended in phosphate-buffered saline (PBS). Each substrate was immersed in the bacterial suspension and incubated at 37 °C for 24 h under static conditions to allow bacterial attachment. The 24 h incubation was used as a standardized endpoint commonly applied for static anti-adhesion/early biofilm assessment, and the resulting images were interpreted as a single-time-point snapshot of bacterial spatial organization [32]. After incubation, the samples were gently rinsed with PBS to remove loosely adhered cells. Adhered bacteria on the six different LIPSS surfaces were assessed by LIVE/DEAD staining. Quantitative analysis was performed on fluorescence images, and the relative bacterial adhesion on each surface was calculated by normalizing to plain SS as the reference (Figure 5c). Here, the single-organism assay was intentionally used to minimize biological variability and to isolate the surface-geometry-driven effect in a controlled, comparative manner [33].

Surfaces with LIPSS exhibited pronounced antifouling performance compared to the non-processed surface. In other words, the introduction of LIPSS effectively suppressed bacterial adhesion, indicating that micro- and sub-microscale surface structuring plays a critical role in regulating bacteria–surface interactions. In particular, a clear dependence of antifouling performance on surface pattern geometry, as shown in Figure 2, was observed. When comparing different pattern shapes, the linear pattern resulted in a lower level of bacterial adhesion (0.0482 to 0.1179; fold change relative to plain SS) than the circular pattern (0.1393 to 0.2442). This finding demonstrates that not only the presence of surface microstructures but also the directionality of the pattern is a decisive factor governing antifouling behavior.

Importantly, as shown in Figure 4, all six LIPSS configurations exhibited nearly identical static water contact angles, indicating that wettability alone cannot account for the observed differences in bacterial adhesion. This allows the antifouling behavior to be clearly attributed to the geometrical features of the laser-textured surface patterns under effectively controlled wettability conditions. These findings collectively establish surface pattern geometry as a key parameter controlling bacterial attachment, highlighting the importance of precise, geometry-driven surface design for intrinsic antifouling functionality.

As shown in Figure 5c, a comparative analysis of laser output intensity revealed that LIPSS fabricated under the 30 mW condition exhibited the most pronounced antifouling effect. At this output level, a well-balanced surface morphology was achieved, leading to maximal inhibition of *E. coli* adhesion (0.0482 for linear/0.1393 for circular fold over plain SS).

In contrast, lower or higher laser intensities resulted in less effective antifouling performance (linear polarization: 0.1179 at lower intensity and 0.0701 at higher intensity, circular polarization: 0.2442 at lower intensity and 0.0701 at higher intensity, fold over plain SS) due to insufficient pattern formation or excessive surface modification (Figure 2). Although increased fs laser output led to noticeable changes in surface microstructure, the strongest suppression of bacterial adhesion was consistently observed at 30 mW.

As discussed in Figure 2, the surface morphology varies with respect to laser output. Specifically, at 17 mW, periodic nanoscale LIPSS are formed, whereas higher outputs in the range of 30 and 45 mW lead to the development of coarser micron-scale features. We believe that these changes could alter the geometric compatibility between the surface texture and the size of *E. coli*, with larger and more irregular features providing more favorable attachment sites [34].

**Figure 6:**
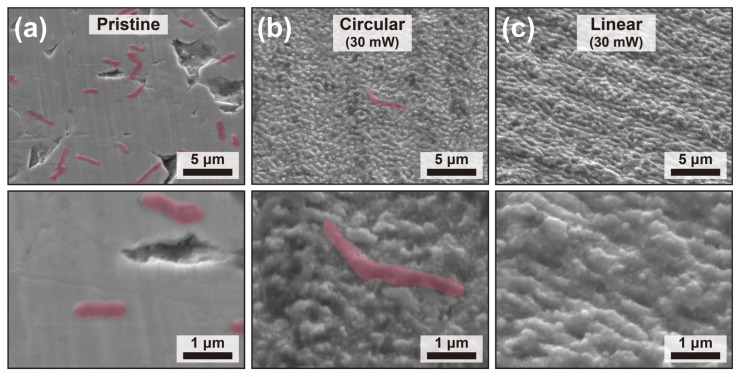
SEM images of *E. coli* adhesion on SS surfaces under different surface conditions. (**a**) Pristine (untreated) SS surface after bacterial seeding, showing a high density of adhered rod-shaped *E. coli* cells distributed across the relatively smooth surface. (**b**) The fs laser–textured SS surface fabricated under circular polarization (30 mW), exhibiting a reduced number of adhered *E. coli* cells with sparse distribution and minimal evidence of clustered adhesion. (**c**) The fs laser–textured SS surface fabricated under linear polarization (30 mW), demonstrating the most pronounced suppression of bacterial adhesion, with only a few isolated *E. coli* cells observed on the surface.

In order to further elucidate the observed antifouling performances in Figure 5, SEM imaging was used to analyze the distribution of *E. coli* cells remaining on SS surfaces after bacterial seeding under different surface conditions (Figure 6), providing direct visual evidence of the antifouling performance of the laser-textured surfaces. Clear differences in bacterial adhesion behavior are observed depending on the surface treatment and laser polarization used during fabrication.

On the pristine (untreated) SS surface (Figure 6a), a large number of rod-shaped *E. coli* cells are observed to adhere uniformly across the relatively smooth surface. The widespread bacterial coverage indicates favorable conditions for bacterial attachment on the untreated SS, where the lack of micro- and nanoscale topographical features allows intimate contact between the bacterial cell body and the substrate.

In contrast, fs laser–textured SS surfaces fabricated under circular polarization with a laser power of 30 mW (Figure 6b) exhibit a reduced number of adhered bacteria. Only sparsely distributed *E. coli* cells are observed, with little evidence of clustered adhesion. This reduction suggests that the introduction of isotropic micro- and nanostructures disrupts stable bacterial attachment by modifying the local surface geometry and reducing the effective contact area between the bacteria and the substrate.

The most pronounced suppression of bacterial adhesion is observed on surfaces textured under linear polarization with a laser power of 30 mW (Figure 6c), where bacterial presence is limited across the examined surface area. This result indicates that linear polarization produces surface morphologies that are particularly effective in inhibiting bacterial attachment, likely due to a synergistic combination of anisotropy and feature density that impedes stable cell–surface interactions.

These SEM observations are consistent with the quantitative bacterial attachment analysis presented in Figure 5c. In particular, linearly polarized laser processing results in lower bacterial attachment compared to circular polarization, in agreement with the reduced bacterial coverage observed in SEM images. The corresponding surface morphologies (Figure 6c) indicate that linear polarization produces anisotropic, linearly aligned LIPSS, whereas circular polarization generates more isotropic protrusions. Based on these observations, we propose that the anisotropic, linearly aligned LIPSS create a geometric mismatch with the rod-shaped *E. coli*, which restricts their orientation and reduces effective contact formation, thereby hindering stable adhesion. This is consistent with prior reports showing that bacterial attachment is strongly influenced by contact geometry and surface topography [35,36]. As a result, bacterial attachment is significantly suppressed. These results clearly demonstrate that both pattern geometry and precise microstructure control of LIPSS are key determinants in the design of effective antifouling surfaces.

Given that this antifouling effect arises from surface-mediated physicochemical interactions rather than organism-specific biochemical inhibition, the underlying design principle observed for *E. coli* is expected to be extendable beyond a single strain, although the magnitude may vary across organisms and contamination conditions. In particular, mixed-species communities and matrix-related effects can override or reshape surface-driven trends, representing an important but distinct experimental dimension [37].

## 3. Discussion

This study demonstrates a high-precision surface modification strategy using fs laser texturing to enhance the bio-resistance of SS. By systematically modulating laser power and polarization, we successfully transitioned surface morphologies from well-defined LIPSS to complex hierarchical textures. A key finding of this research is the decoupling of antifouling performance from surface wettability; despite varying morphologies, the textured surfaces maintained consistent water contact angles (32.6–36.9°). This suggests that the significant inhibition of *E. coli* colonization, confirmed via fluorescence microscopy and SEM, is primarily driven by the geometric mismatch arising from the specific spatial arrangement and anisotropy of the textures. Linear laser-induced textures exhibited fundamentally different bacterial inhibition behavior compared to circular patterns, despite comparable wettability, underscoring laser-pattern geometry as an independent parameter for bio-resistant surfaces. Furthermore, unlike coating-based approaches susceptible to delamination, the intrinsic and monolithic nature of the laser-textured surfaces suggests superior robustness suitable for harsh environments [19,38]. As a chemical-free and monolithic physical modification, the fs laser texturing approach provides a robust framework for the strategic design of mechanically durable, bio-resistant metallic surfaces in various industrial applications and high-precision surface engineering.

## 4. Materials and Methods

Materials: Stainless steel substrates (AISI 301, Goodfellow, Pittsburgh, PA, USA) with 0.5 mm thickness were used as target specimens. Bacterial LIVE/DEAD staining kit was purchased from Invitrogen (Carlsbad, CA, USA).

Femtosecond laser processing: A 500-fs laser (1030 nm, 100 kHz; s-Pulse, Amplitude, Pessac, France) was synchronized with motorized XYZ translation stages (PI-USA) to enable precise beam–sample alignment and patterning. The laser beam was focused onto the sample using a 5× near-infrared objective lens (Mitutoyo, Kawasaki, Japan), yielding a spot diameter of approximately 16 μm. Incident laser power was controlled by a combination of a polarizing beam splitter and a half-wave plate, allowing fine adjustment of the delivered fluence.

Contact angle measurements: Static contact angles were measured using the sessile drop method. A droplet of ultrapure water was gently deposited onto the substrate surface using a microsyringe. Side-view images of the droplet were captured within 5 s after deposition to minimize evaporation and dynamic spreading effects. The acquired images were analyzed using ImageJ software (Fiji distribution, version 2.9.0, National Institutes of Health, Bethesda, MD, USA).

Antifouling test on LIPSS substrate: The antifouling properties of the substrates were assessed using *Escherichia coli* (*E. coli*) as a representative bacterial strain. *E. coli* was grown in Luria–Bertani broth at 37 °C for 16 h with shaking at 160 rpm using a rotary incubator (Incu-Shaker Mini, Benchmark Scientific, Sayreville, NJ, USA). After cultivation, the bacterial culture was centrifuged at 4000 rpm for 5 min to harvest the cells. The supernatant was removed, and the cell pellet was resuspended in phosphate-buffered saline (PBS). For the bacterial adhesion test, the substrates were completely submerged in the bacterial suspension and incubated under static conditions at 37 °C for 24 h. Following incubation, the samples were gently washed three times with physiological saline to eliminate loosely attached bacteria. The adhered bacteria on the substrates were stained using a LIVE/DEAD BacLight bacterial viability kit (Invitrogen, USA) in accordance with the manufacturer’s protocol. Fluorescence images of the stained *E. coli* were obtained using a fluorescence microscope (Keyence, Itasca, IL, USA). All images were cropped to an identical area, and bacterial adhesion was quantitatively evaluated by image analysis using ImageJ software (National Institutes of Health, USA).

Surface morphology characterization: Scanning electron microscopy (JEOL) was used to examine the surface morphology.

## Figures and Tables

**Table 1:** Summary of laser processing parameters used for the fabrication of hierarchical SS surfaces.

Parameter	Value	Unit	Type
Pulse duration	500	fs	Fixed
Wavelength	1030	nm	Fixed
Repetition rate	100	kHz	Fixed
Spot diameter	16	μm	Fixed
Line spacing	2	μm	Fixed
Scanning speed	5	mm/s	Fixed
Pulse overlap	99.7	%	Fixed
Number of scans	1	-	Fixed
Power	17, 30, 45	mW	Variable
Fluence	0.085, 0.149, 0.224	J/cm^2^	Variable
Polarization	Linear, Circular	-	Variable

## Data Availability

The authors declare that the data supporting the findings of this study are available within the article.

## Abbreviations

FS	Femtosecond
SS	Stainless steel
LIPSS	Laser-induced periodic surface structures
SEM	Scanning electron microscopy
*E. coli*	*Escherichia coli*
